# *N*^6^-methyladenosine and Its Implications in Viruses

**DOI:** 10.1016/j.gpb.2022.04.009

**Published:** 2022-07-11

**Authors:** Yafen Wang, Xiang Zhou

**Affiliations:** College of Chemistry and Molecular Sciences, Wuhan University, Wuhan 430072, China

**Keywords:** m^6^A, Writer protein, Reader protein, Eraser protein, Virus

## Abstract

*N*^6^-methyladenine (**m^6^A**) is the most abundant RNA modification in mammalian messenger RNAs (mRNAs), which participates in and regulates many important biological activities, such as tissue development and stem cell differentiation. Due to an improved understanding of m^6^A, researchers have discovered that the biological function of m^6^A can be linked to many stages of mRNA metabolism and that m^6^A can regulate a variety of complex biological processes. In addition to its location on mammalian mRNAs, m^6^A has been identified on viral transcripts. m^6^A also plays important roles in the life cycle of many viruses and in viral replication in host cells. In this review, we briefly introduce the detection methods of m^6^A, the m^6^A-related proteins, and the functions of m^6^A. We also summarize the effects of m^6^A-related proteins on viral replication and infection. We hope that this review provides researchers with some insights for elucidating the complex mechanisms of the epitranscriptome related to viruses, and provides information for further study of the mechanisms of other modified nucleobases acting on processes such as viral replication. We also anticipate that this review can stimulate collaborative research from different fields, such as chemistry, biology, and medicine, and promote the development of antiviral drugs and vaccines.

## Introduction

Gene expression and cell growth are not only controlled by genetics but also closely related to epigenetic regulation, such as chemical modifications of RNA. The term “RNA modifications” refers to various forms of modifications that occur on RNA, which are called epitranscriptomic modifications. It is worth mentioning that the term “epitranscriptomics” is still controversial. More than 160 types of post-transcriptional modifications have been found in archaea, bacteria, viruses, and eukaryotes [1], and these modifications are widely present on various RNA types, such as messenger RNA (mRNA), transfer RNA (tRNA), ribosomal RNA (rRNA), microRNA (miRNA), and long non-coding RNA (lncRNA). RNA modifications play important roles in the regulation of RNA processing, are important components of post-transcriptional regulation, and can affect the secondary structure, splicing, stability, and translation of RNA. These modifications further regulate important physiological and pathological processes such as embryo development, tissue and organ differentiation, long-term memory formation, and tumorigenesis [2], [3], [4]. Among RNA modifications, *N*^6^-methyladenine (m^6^A) is the most widely distributed form of methylation on eukaryotic RNAs [5], [6], and is also the most thoroughly studied type of RNA modifications. m^6^A was first identified half a century ago as a potential regulator of intracellular mRNA processing. A study conducted in the 1970s discovered that m^6^A also exists on viral transcripts [7]. m^6^A has been identified in a number of viruses, including human immunodeficiency virus (HIV), Rous sarcoma virus, herpes simplex virus 1 (HSV-1), adenovirus, simian virus 40 (SV40), and endogenous retroviruses [7], [8], [9], [10], [11], [12], [13], [14], [15], [16]. In addition to influencing cellular physiological processes, m^6^A plays roles in viral processes, such as the regulation of viral RNA replication and immune processes [17], [18], [19]. Here, we summarize the writer, reader, and eraser proteins and describe the mechanism of action of m^6^A during viral infection. For example, we provide an overview of recent studies showing that m^6^A regulates the replication and infection of severe acute respiratory syndrome coronavirus 2 (SARS-CoV-2) and host interaction processes. We hope that epigenetic modifications will provide new insights into the development of vaccines and antiviral drugs.

## Distribution of m**^6^**A

Since the identification of the first modified base, pseudouridine, a variety of RNA modifications have successively been found, and m^6^A is the most abundant and one of the most thoroughly studied base modifications. In 1974, researchers used isotope labeling to study mRNA methylation in mammalian cells and detected the presence of m^6^A [20], [21]. In mammals, m^6^A is found on approximately 0.1%–0.4% of all adenosines in RNA, and each mRNA has approximately three m^6^A sites [19], [22]. m^6^A is mainly concentrated in near-stop codon regions and 3′-untranslated regions (3′-UTRs). Some organisms and organs also contain m^6^A near the 5′-UTR and start codons [23], [24], [25], [26]. m^6^A is usually found in the conserved DRACH (D = A, G, U; R = G, A; H = A, C, U) sequence [27]. As m^6^A has gained widespread attention, it has been found in a variety of species, such as mice [28], zebrafish [29], *Arabidopsis* [30], [31], rice, potato [32], and viruses [17], [33], [34], [35]. The distribution of m^6^A modifications in different species is highly conserved. For example, approximately half of m^6^A sites are identical between human embryonic stem cells (ESCs) and mouse ESCs [36].

## m^6^A writer, eraser, and reader proteins

m^6^A is formed by the addition of a methyl group to the amino group at the 6-position of adenosine by a methyltransferase. The first key methyltransferase, METTL3, was discovered as early as in 1994 [37], and subsequently, another protein in the METTL family — METTL14, which also has methyltransferase activity — was also identified as an important component of the methyltransferase complex [38], [39]. METTL3 and METTL14 form a 1:1 stable heterodimer and enhance methyltransferase activity through a synergistic action, in which METTL3 acts as the active catalytic subunit and METTL14 enhances substrate recognition and RNA binding. The knockout of *METTL3* or *METTL14* decreases the m^6^A content [40], [41], which indicated that METTL3 and METTL14 play a role in the formation of m^6^A. However, other studies have shown that the main function of METTL14 is not to catalyze methyl transfer but to provide an RNA-binding scaffold to activate and enhance the catalytic activity of METTL3 [38]. WTAP is also an active component of the methyltransferase complex [26], [39], [42], but unlike METTL3, WTAP has no methyltransferase activity. The knockout of *WTAP* decreases the binding of METTL3 to RNA and the m^6^A content [42]. WTAP can bind to the METTL3–METTL14 complex such that the complex is concentrated in nuclear spots to enhance mRNA methylation. Several other methyltransferases or enzymes with the ability to recruit methyltransferase complexes to target genes have also been identified, and these include METTL5 [43], METTL16 [44], KIAA1429 [45], RBM15, RBM15B [46], HAKAI, ZC3H13 [47], and ZCCHC4 [48].

The opposite of methylase activity is demethylase activity. He et al. found that the knockdown and overexpression of *FTO* increases and decreases the m^6^A content, respectively [49]. FTO, an obesity-related protein and a dioxygenase based on α-ketoglutarate [50], has the same conserved functional domain as AlkB, a demethylase family protein [51]. Written m^6^A is erased by FTO and reverts to adenosine, and this finding provides the first demonstration that m^6^A is dynamically modified and starts a new chapter in the understanding of m^6^A. FTO exhibits demethylase activity not only *in vivo* but also toward single-stranded DNA and RNA *in vitro*. FTO is also involved in tumorigenesis (*e.g.*, by acting as an oncogene in acute myeloid leukaemia [52]) and can modulate plant growth (*e.g.*, by increasing the rice yield and potato biomass) [32]. Notably, FTO can also demethylate *N*^6^,2′-*O*-dimethyladenosine (m^6^Am) [53].

In addition to FTO, ALKBH5 in the AlkB family has the function of erasing methylation [54]. The knockout and overexpression of *ALKBH5* in cells are accompanied by an increase and a decrease in the m^6^A content, respectively. *ALKBH5* knockdown promotes the transport of mRNA from the nucleus to the cytoplasm and affects the metabolism of RNA and the assembly of mRNA processing factors. In ALKBH5-deficient male mice, the apoptosis of spermatocytes during meiotic metaphase is affected, which leads to impaired fertility. ALKBH5 also functions in the maintenance of tumorigenicity, self-renewal, and tumorigenesis in glioma [55], [56], promotes the radioresistance and invasion of glioma stem cells [57], and suppresses tumor progression [58]. These results indicate that dynamic changes in m^6^A play an important role in regulating various biological activities.

Following the discovery of the aforementioned m^6^A “writer” and “eraser” proteins, the m^6^A-binding proteins that “read” m^6^A were also discovered. YT521-B homology (YTH) domains can bind to m^6^A-modified RNA [59]. The knockout of *YTHDF2* increases the expression levels of m^6^A-containing mRNAs, which indicates that YTHDF2 can affect the stability of mRNA by binding to m^6^A [60]. Another m^6^A reader protein, YTHDF1, promotes protein synthesis by interacting with initiation factors and ribosomes [61], and YTHDF3 can cooperate with YTHDF1 to promote protein synthesis. YTHDF1–3 can affect metabolism by binding to m^6^A-containing mRNAs [62], [63]. IGF2BP1–3 have also been identified as m^6^A reader proteins. Unlike recognition by YTHDF2, which reduces the stability of m^6^A-containing mRNAs, the recognition of m^6^A-containing mRNAs by IGF2BP enhances mRNA stability and translation [64]. The m^6^A-related proteins are summarized in Table 1.

**Table 1:** **Roles of m^6^A-related proteins**

**Type**	Protein **name**	**Function**
Writer	METTL3 METTL14 WTAP KIAA1429 RBM15 RBM15B HAKAI ZC3H13 ZCCHC4	The METTL3–METTL14 complex catalyzes the adenosine methylation of RNA to form m^6^A; other proteins are important components of the complex
Eraser	FTO ALKBH5	Mediate m^6^A demethylase
Reader	YTHDF1 YTHDF2 YTHDF3 IGF2BP1 IGF2BP2 IGF2BP3	Bind to m^6^A sites; increase or decrease mRNA stability; regulate RNA processing

*Note*: METTL3, methyltransferase-like 3; METTL14, methyltransferase-like 14; WTAP, Wilms’ tumor 1-associating protein; RBM15, RNA binding motif protein 15; RBM15B, RNA binding motif protein 15B; ZC3H13, zinc finger CCCH-type containing 13; ZCCHC4, zinc finger CCHC-type containing 4; FTO, fat mass and obesity-associated protein; ALKBH5, alkB homologue; YTHDF1, YTH domain family protein 1; YTHDF2, YTH domain family protein 2; YTHDF3, YTH domain family protein 3; IGF2BP1, insulin like growth factor 2 mRNA binding protein 1; IGF2BP2, insulin like growth factor 2 mRNA binding protein 2; IGF2BP3, insulin like growth factor 2 mRNA binding protein 3.

## Functions of m**^6^**A

High-throughput sequencing and quantitative polymerase chain reaction (qPCR) analysis have revealed that the abundance of m^6^A differs among different types of mRNA. Approximately 46% of mRNAs contain only one m^6^A peak, 37.3% of mRNAs contain two m^6^A peaks, and a few mRNAs contain at least three m^6^A peaks [25]. The discovery of m^6^A-related proteins has led to the gradual discovery of the related functions of m^6^A and its role in life processes. For example, m^6^A is involved in RNA processing, development, differentiation, metabolism, and fertility. The process of eukaryotic mRNA formation is divided into transcription, splicing, export, translation, and degradation, and studies have shown that m^6^A affects the life cycle of mRNA [65].

### mRNA splicing

The processing of a precursor RNA to a mature mRNA requires 5′-capping, 3′-polyadenylation, intron excision, and exon splicing. The precise excision of introns and the splicing of exons are key factors in gene expression that directly affect the diversity of proteins. m^6^A is more abundant in RNA precursors than in mature mRNA [66]. Changes in the expression of WTAP or METTL3 can regulate the expression of related genes and splicing subtypes in transcription and RNA processing, which indicates that writer proteins can affect RNA splicing [42]. In addition, m^6^A reader proteins can interact with other splicing factors to regulate splicing. Most of the m^6^A-related proteins are located in nuclear spots enriched with splicing factors [54], [67], which facilitate the connection between m^6^A and RNA splicing. The splicing efficiency is also important for coordinating gene expression, and the deposition of m^6^A on transcripts near the splice junction increases the splicing efficiency [68].

### mRNA export from the nucleus

The export of transcribed mRNAs from the nucleus to the cytoplasm is an important process in mRNA translation [69]. *METTL3* silencing leads to significant nuclear accumulation and delayed transcript processing [70]. Similarly, the deletion of *YTHDC1* prolongs the retention time of mRNA in the nucleus, which allows the accumulation of transcripts in the nucleus [71]. In contrast, the deletion of *ALKBH5* induces the nuclear export of mRNA [54]. Thus, m^6^A can regulate the process of mRNA export from the nucleus to the cytoplasm and further affect the process of gene expression.

### mRNA translation

METTL3 can promote the translation of mRNAs such as the epidermal growth factor receptor and the Hippo pathway effector TAZ. The depletion of METTL3 reduces the translation efficiency, whereas both wild-type and non-methylated METTL3 promote translation. Mechanistic studies have shown that METTL3 enhances mRNA translation through its interaction with the translation initiation machinery independent of its intrinsic activity [72]. YTHDF1 can not only promote the binding of m^6^A-containing mRNAs to ribosomes but also recruit translation initiation factor complex 3 (eIF3) to promote mRNA translation [61]. m^6^A can also promote mRNA translation that is resistant to eIF4 inactivation [73].

### mRNA stability

The last step in mRNA metabolism is mRNA degradation, and the stability of an mRNA is closely related to its degradation. Specifically, mRNA stability is regulated by m^6^A; for example, the stability of transcripts is increased in METTL3- or METTL14-depleted cells [74]. YTHDF2 binds to m^6^A-containing mRNA and transports this mRNA to the site of mRNA degradation in the cytoplasm, which accelerates the degradation of mRNA. However, not all m^6^A reader proteins reduce mRNA stability. For example, IGF2BPs can enhance mRNA stability [64]. ALKBH5 affects the meiotic and haploid stages of spermatogenesis by controlling the splicing and stability of mRNAs [75].

## Methods for m**^6^**A detection

**Figure 1:**
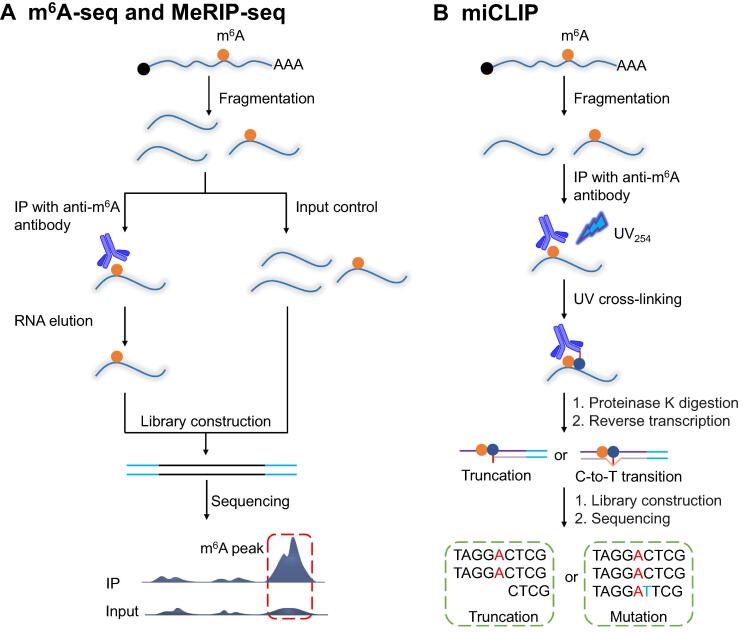
**Schematic diagram of m^6^A detection method** **A.** Schematic diagram of m^6^A-seq [24]. The fragmented mRNA is enriched with m^6^A-antibody, and non-enriched RNA is used as control. The information containing m^6^A is obtained by comparing the sequenced fragments before and after enrichment with antibody. **B.** Schematic diagram of miCLIP [27]. After the fragment mRNA was enriched with antibodies, UV cross-linking was carried out, and misincorporation or reverse transcription termination would occur near the cross-linking sites during reverse transcription. m^6^A, *N*^6^-methyladenine; IP, immunoprecipitation; m^6^A-seq, *N*^6^-methyladenine sequencing; MeRIP-seq, methylated RNA immunoprecipitation sequencing; miCLIP, m^6^A individual-nucleotide-resolution cross-linking and immunoprecipitation; UV, ultraviolet.

To date, a large number of methods for the detection of m^6^A have been reported. Because the methods described in below are mainly based on the strategy of m^6^A-specific antibody enrichment, we briefly introduce these technologies. m^6^A-specific antibodies are a widely used m^6^A detection method; these antibodies can specifically bind to m^6^A-containing RNAs and enrich these RNAs for analysis, which allows m^6^A immunoprecipitation sequencing, *N*^6^-methyladenine sequencing (m^6^A-seq) or methylated RNA immunoprecipitation sequencing (MeRIP-seq) (Figure 1A) [24], [25]. In MeRIP-seq, mRNA is first cleaved into short fragments ranging from 100 nt to 200 nt, and the transcriptome-wide m^6^A distribution profile can be obtained by specific m^6^A antibody binding, enrichment, and high-throughput sequencing. However, because the resolution of m^6^A peaks obtained with the antibody binding approach is 100–200 nt, the accurate localization of m^6^A marks is impossible, and various strategies have thus been developed to improve the resolution. A novel m^6^A photocrosslinking sequencing method (PA-m^6^A-seq) was developed by introducing the modified base to improve the resolution to 23 nt [76]. Ultraviolet (UV) radiation-induced crosslinking coupled with immunoprecipitation sequencing (CLIP-seq) can reveal interactions between RNA and RNA-binding proteins at the genome-wide level. CLIP-seq and MeRIP-seq were combined to develop a novel m^6^A-specific UV crosslinking immunoprecipitation sequencing technology (miCLIP) (Figure 1B) [27], which achieves m^6^A sequencing at single-base resolution. This strategy can also provide m^6^A information beyond the conserved RRACH sequence, which compensates for the deficiency of MeRIP-seq. The aforementioned methods cannot be used for the quantitative analysis of m^6^A and the determination of the m^6^A modification level in different transcripts of the same gene. However, m^6^A-level and isoform-characterization sequencing (m^6^A-LAIC-seq) [77] allows the quantitative analysis of m^6^A levels in specific gene transcripts based on MeRIP-seq. Because m^6^A antibodies can also recognize m^6^Am, the antibody-based methods described above cannot distinguish m^6^A from m^6^Am. *N*^6^,2′-*O*-dimethyladenosine-sequencing (m^6^Am-seq) is a recently reported method that effectively distinguishes m^6^A from m^6^Am [78]. In addition to antibody enrichment, the single-base-resolution detection of m^6^A can be achieved using restriction endonuclease methods [79], [80], but these restriction endonuclease methods have sequence biases. More information about m^6^A can be found in some of the latest reviews [81], [82].

## Relationship between m**^6^**A and viruses

m^6^A is present not only on cellular RNA but also on viral mRNA and regulates the expression levels of viral genes, which are closely related to the viral life cycle. Studies conducted in the 1970s discovered that m^6^A is present on the RNA transcripts of some viruses, such as influenza A virus, SV40, Rous sarcoma virus, and adenovirus. Researchers have speculated that m^6^A plays a role in regulating viral infection, but the exact mechanism and function were unclear. It was not until the 21st century that m^6^A was revealed to have functions such as regulating viral replication. Further research has shown that m^6^A is found in many types of viruses. The effects of m^6^A-related proteins on some viruses are briefly introduced in this section (Table 2).

**Table 2:** **Functions of m^6^A-related proteins in viruses**

**Virus**	**Gene name**	**Expression level**	**Consequence**	**Ref.**
SV40	*METTL3*	Down	Reduced viral replication	[85]
	*YTHDF2*	Up	Enhanced viral replication; larger viral plaques	
	*YTHDF2*	Down	Reduced viral replication	
HBV	*METTL3*, *METTL14*	Down	Increased HBV protein expression	[87]
	*YTHDF2*, *YTHDF3*	Down	Increased HBV protein expression	
	*FTO*, *ALKBH5*	Down	Decreased HBV protein expression	
KSHV	*METTL3*	Down	Abolished lytic gene expression (BCBL1 cells)	[91]
	*FTO*	Down	Enhanced lytic gene expression (BCBL1 cells)	
	*METTL3*	Down	Increased protein expression (TREX-BCBL-1 cells); reduced protein levels (iSLK.219 cells)	[94]
	*YTHDF2*	Down	Increased protein expression (iSLK.BAC16 cells)	
	*YTHDF2*	Up	Reduced viral production; decreased levels of viral proteins (endothelial cells)	[93]
	*YTHDF1*, *YTHDC1*, *YTHDC2*	Down	No significant or consistent effect on viral lytic replication	
	*YTHDF3*	Down	Reduced viral lytic replication (endothelial cells)	
HIV-1	*METTL3*, *METTL14*	Down	Decreased viral replication	[97]
	*ALKBH5*	Down	Increased viral replication	
	*YTHDF2*	Up	Enhanced replication	[95]
	*YTHDF2*	Down	Reduced replication	
	*YTHDF1*, *YTHDF2*, *YTHDF3*	Down	Enhanced infection	
	*YTHDF1*, *YTHDF2*, *YTHDF3*	Up	Decreased viral genomic RNA levels	[99]
	*YTHDF1*, *YTHDF2*, *YTHDF3*	Down	Increased viral infectivity	
ZIKV	*METTL3*, *METTL14*	Up	Decreased viral titer	[100]
	*METTL3*, *METTL14*	Down	Increased viral production	
	*YTHDF1*, *YTHDF2*, *YTHDF3*	Up	Decreased ZIKV RNA expression	
	*YTHDF1*, *YTHDF2*, *YTHDF3*	Down	Increased viral replication	
	*ALKBH5*	Up	Increased viral titer	
	*ALKBH5*, *FTO*	Down	Decreased viral production	
HCV	*METTL3*, *METTL14*	Down	Increased infectious HCV particle production	[101]
	*YTHDF1*, *YTHDF2*, *YTHDF3*	Down	Increased infectious HCV particle production	
	*FTO*	Down	Decreased infectious HCV particle production	
SARS-CoV-2	*METTL3*, *METTL14*	Down	Increased viral replication	[106]
	*YTHDF2*	Down	Enhanced viral infection and replication	
	*ALKBH5*	Down	Decreased viral replication	

*Note*: SV40, simian virus 40; HBV, hepatitis B virus; KSHV, Kaposi’s sarcoma-associated herpesvirus; HIV-1, human immunodeficiency virus-1; ZIKV, Zika virus; HCV, hepatitis C virus; SARS-CoV-2, severe acute respiratory syndrome coronavirus 2.

### m**^6^**A in SV40

SV40, which belongs to the polyomavirus family and is an oncogenic virus found in both humans and monkeys, is the first animal virus whose complete genomic DNA sequence has been determined. The SV40 genome is a circular double-stranded DNA with a size of 5.2 kb. Researchers discovered the presence of m^6^A in SV40 mRNA more than 40 years ago [83], and subsequent studies revealed that m^6^A in pre-mRNA is very important for the generation of late SV40 mRNAs [84]. Regulation of the m^6^A content in the cytoplasm of SV40-infected best supportive care-1 (BSC-1) cells revealed that internal m^6^A plays a role in regulating the processing and export of mRNA from the nucleus to the cytoplasm in non-transformed cells. Cullen’s research group revealed that m^6^A can positively regulate SV40 gene expression [85]. The overexpression of *YTHDF2* in SV40-infected BSC40 cells results in faster viral replication and larger virus plaques. The mutational inactivation of YTHDF2 and METTL3 yields opposite results. Photoactivatable ribonucleoside-enhanced CLIP (PAR-CLIP) [86] and PA-m^6^A-seq [76] identified two m^6^A peaks in the early SV40 region and 11 m^6^A peaks in the late transcript. The reduction of m^6^A in late mRNAs slows the replication speed of viral mutants, whereas a decrease in m^6^A in early-stage mRNAs has no effect on infection. The deletion of m^6^A in late mRNAs prevents nuclear processing and reduces the expression of the encoded structural protein VP1, which indicates that m^6^A affects the translation of late mRNAs. The mechanism by which m^6^A affects the nucleation of late mRNAs needs further study.

### m**^6^**A in hepatitis B virus

The hepatitis B virus (HBV) genome is very small, containing approximately 3200 nucleotides, and HBV completes its life cycle through replicative intermediate pregenomic RNA (pgRNA). Siddiqui et al. revealed how m^6^A regulates HBV gene expression and the reverse transcription of pgRNA [87]. These researchers first identified the presence of m^6^A on HBV transcripts by MeRIP-seq. The silencing of *METTL3* and *METTL14* (1) decreased the level of m^6^A on HBV pgRNA, (2) reduced the reverse transcription of pgRNA, and (3) increased the expression of HBV protein. MeRIP-seq showed that the typical DRACH motif for m^6^A in the HBV epsilon loop contains m^6^A at the A1907C site. The epsilon loop is located at the end of HBV mRNAs and the 5′ and 3′ ends of pgRNAs. The mutation of m^6^A in the pgRNA 5′ epsilon loop blocks the reverse transcription of pgRNA, and the loss of m^6^A in the 3′ epsilon loop increases the half-life of HBV. These results indicate that m^6^A in the 5′ stem loop positively regulates reverse transcription, whereas m^6^A in the 3′ stem loop negatively regulates RNA stability. These studies show that m^6^A has a dual regulatory function in HBV. m^6^A can more finely tune the events in the HBV life cycle, and this finding improves the understanding of the life cycle of HBV.

Subsequently, Siddiqui et al. reported that m^6^A can regulate host innate immunity to HBV infection [88]. The depletion of METTL3 and METTL14 increases the retinoic acid-inducible gene I (RIG-I) recognition of viral RNA, which further stimulates the production of type I interferons. The opposite results have been found in *METTL3* and *METTL14* overexpression systems. An increase in the m^6^A content decreases mRNA stability and thus regulates the translation level and abolishes the innate immune response. The binding of YTHDF2 to m^6^A-modified viral RNA blocks the recognition of viral RNA by RIG-I, and thus, HBV can achieve immune evasion through m^6^A.

ISG20 is an exonuclease that binds to and degrades HBV transcripts. Siddiqui et al. found that ISG20 is an interaction partner of YTHDF2, and the resulting complex recognizes m^6^A-containing HBV RNA and performs exonuclease activity [89]. The silencing of *METTL3* and *METTL14* or *YTHDF2* produces HBV transcripts that are resistant to interferon-α (IFN-α) treatment or ISG20-mediated degradation. Mutation of the m^6^A residue A1907 revealed that this m^6^A site is a key factor in IFN-α-mediated HBV RNA decay, and these results provided the first demonstration of the role of m^6^A in IFN-α-induced viral RNA degradation.

### m**^6^**A in Kaposi’s sarcoma-associated herpes virus

Kaposi’s sarcoma-associated herpes virus (KSHV) is associated with some malignancies, including primary effusion lymphoma (PEL), Kaposi’s sarcoma (KS), and multicentric Castleman’s disease (MCD) [90]. The KSHV replication cycle consists of latent and lytic replication phases, which are important for the development of KSHV-related cancers. Nilsen et al. found that KSHV can use m^6^A to regulate its lytic replication [91]. These researchers first identified m^6^A on most KSHV transcripts through MeRIP-seq. The stimulation of lytic replication increases the level of m^6^A-containing viral mRNA. *FTO* knockdown increases the expression of lytic genes, whereas *METTL3* knockdown decreases the expression of lytic genes. Researchers have used two small-molecule inhibitors and obtained consistent results. Meclofenamic acid can selectively inhibit FTO, and 3-deazaadenosine (DAA) can inhibit the hydrolysis of *S*-adenosylhomocysteine ​​(SAH) to block the formation of m^6^A. Researchers have found that the addition of meclofenamic acid increases the expression of cleavage genes and that the blockade of m^6^A formation by DAA abolishes the expression of cleavage genes. Replication transcription activator (RTA) is an important KSHV cleavage switch protein, and its pre-mRNA contains m^6^A. The blockade of m^6^A formation inhibits splicing of the RTA pre-mRNA and then terminates viral lytic replication. The polyadenylated nucleus (PAN) is a long non-coding transcript involved in the cleavage of KSHV and viral gene expression. Sztuba et al. found that m^6^A on PAN varies dynamically and that modification is increased at the late cleavage stage of KSHV infection [92]. These studies suggest that m^6^A regulates the replication of KSHV cleavage genes.

Gao et al. also found that most KSHV transcripts are methylated during lytic replication [93]. The knockdown of *YTHDF2* increases KSHV cleavage replication, and the overexpression of *YTHDF2* reduces the production of virus and the level of viral proteins. These findings are obtained because the binding of YTHDF2 to viral transcripts affects the stability of viral RNA and suggest that YTHDF2 may mediate a host defence mechanism in which the degradation of KSHV is regulated through YTHDF2 and that KSHV cleavage replication is then regulated to inhibit viral replication. Glaunsinger et al. reported that infection with KSHV significantly increases the level of m^6^A in host cells [94]. Interestingly, m^6^A exerts different proviral and antiviral effects on viral gene expression in different cell types. In some cell types, the knockdown of *METTL3* and *YTHDF2* suppresses the production of virus particles, and m^6^A functions in a proviral manner. The viral lytic transactivator gene *ORF50* contains m^6^A, and this m^6^A positively regulates *ORF50*. In other cell types, the knockdown of *METTL3* and *YTHDF2* exerts antiviral effects. These findings suggest that m^6^A can regulate KSHV gene expression and that this effect can yield significantly different results in different cells.

### m**^6^**A in HIV-1

HIV-1 can attack the human immune system, particularly the most important CD4^+^ T lymphocytes in the human body, and destroying a large number of CD4^+^ T cells results in loss of human immune function. Therefore, patients infected with HIV are susceptible to a variety of diseases and malignant tumors and have a higher mortality rate than the general population. Cullen et al. used PA-m^6^A-seq to identify the presence of m^6^A in the HIV-1 genome. The m^6^A sites are concentrated in the 3′-UTR and enhance mRNA expression through the recruitment of YTHDF [95]. In CD4^+^ T cells, *YTHDF* overexpression increases HIV-1 protein expression and viral replication, and the opposite effects are observed in *YTHDF*-knockout cells. This finding shows that m^6^A and YTHDF can positively regulate the expression of HIV-1 mRNA. Researchers have also demonstrated that increased m^6^A levels can enhance HIV-1 replication due to the nuclear m^6^A reader YTHDC1 and the cytoplasmic m^6^A reader YTHDF2 [96].

Rana et al. found that the m^6^A content in both the virus and host genomes in HIV-1-infected CD4^+^ T cells is increased [97]. The knockdown of *METTL3* or *METTL14* by short hairpin RNAs (shRNAs) significantly reduces viral replication, and increased reduction is obtained with the knockdown of both *METTL3* and *METTL14*. In contrast, the knockdown of *ALKBH5* significantly increases viral replication. These results indicate that enzymes associated with m^6^A can affect HIV-1 replication. The presence of methylation at A7877 and A7883 in the HIV-1 Rev response element (RRE) RNA stem loop II region was identified by MeRIP-seq, and methylation at these two sites enhances the binding of the HIV-1 Rev protein to the RRE. Mutation of A7883 in the RRE bulge region results in a significant decrease in viral replication and severely affects the nuclear export of viral RNA.

Wu et al. reported that the binding of YTHDF1–3 to m^6^A-modified mRNA reduces HIV-1 reverse transcription and thereby inhibits HIV-1 infection [98]. This research group found that *YTHDF1–3* overexpression reduces the HIV-1 genomic RNA (gRNA) levels and inhibits early and late reverse transcription of gRNA. Changes in two m^6^A sites on the 5′ leader sequence in gRNA reduces the infectivity of HIV-1; in other words, m^6^A changes the infectivity of the virus. The knockdown of *YTHDF1* or *YTHDF3* increases the infectivity of HIV-1 [99]. These results suggest a new mechanism by which m^6^A participates in the regulation of HIV-1 replication and viral interaction with the host immune system.

### m**^6^**A in Flaviviridae

The family Flaviviridae includes a variety of viruses, such as hepatitis C virus (HCV), Zika virus (ZIKV), and dengue virus. Rana et al. found that the m^6^A content of ZIKV RNA is regulated by host methyltransferases and demethylases [100]. ZIKV infection can change the location of m^6^A on mRNA, the motif of m^6^A, and the target genes of methyltransferases in the host. ZIKV replication increases after *YTHDF* silencing. *METTL3* and *METTL14* knockdown increases ZIKV production, whereas the silencing of *FTO* and *ALKBH5* decreases ZIKV production. The overall effect of m^6^A on viral replication may be due to the regulation of viral RNA metabolism by the binding of YTHDF to m^6^A-containing viral RNA.

Horner et al. demonstrated that m^6^A also regulates HCV infection and that m^6^A-related enzymes affect the HCV life cycle [101]. Methyltransferase depletion does not affect the replication of HCV RNA but promotes the production of infectious virus particles to increase the HCV infection rate. The depletion of FTO reduces the infection rate of HCV. Unlike the results obtained for other viruses, in which m^6^A regulates viral replication by regulating the stability or translation of other viral RNAs, m^6^A regulates the production of infectious virus particles through the interaction of HCV RNA with host and viral proteins and thereby regulates the HCV infection rate. Researchers have also found that infection with Flaviviridae family members alters the m^6^A content in the transcripts of certain cells. For example, changes in the m^6^A content in these transcripts during viral infection can affect translation or alternative splicing [102]. These studies lay the foundation for studying the influence of m^6^A on Flaviviridae infection and pathogenesis.

### m**^6^**A in SARS-CoV-2

SARS-CoV-2 is a positive-sense, single-stranded RNA virus with a genome size of 30 kb [103]. SARS-CoV-2 has caused a global health emergency since its initial outbreak in 2020. Dozens of RNA modification sites have been identified on the RNA of SARS-CoV-2 by nanopore sequencing [104]. Yang et al. identified 13 m^6^A peaks on SARS-CoV-2 using MeRIP-seq [105]. Viral subgenomic mRNA (sgRNA) with a regular 3′-UTR can be methylated by host METTL3, which activates the cellular degradation program to remove the viral RNA. To protect against m^6^A-dependent degradation, the m^6^A-modified RRACH motif is eliminated in SARS-CoV-2 to form a shorter 3′-UTR, and this shorter 3′-UTR prevents viral RNA degradation. Therefore, m^6^A may potentially regulate the abundance of SARS-CoV-2 RNA. These speculations need to be further studied in more complete animal models.

Qin et al. performed the first systematic analysis of the m^6^A profile of the SARS-CoV-2 transcriptome and confirmed that m^6^A in the SARS-CoV-2 genome is dynamically modified in human and monkey cells [106]. METTL3/14 and ALKBH5 negatively and positively regulate SARS-CoV-2 replication, respectively. SARS-CoV-2 infection also changes the host m^6^A methylome, which indicates that m^6^A is involved in the host–virus interaction. These findings provide ideas for the development of new antiviral drugs based on m^6^A.

Rana et al. showed that m^6^A plays a role in evasion of the host immune response to SARS-CoV-2 infection. The absence of METTL3 increases the binding of RIG-I, which enhances the innate immune signaling pathway and inflammatory gene expression [107]. Through the indirect inhibition of METTL3 to disrupt the viral life cycle and the direct regulation of the level of m^6^A in the viral genome to enhance the comprehensive effect of a timely innate immune response, METTL3 is expected to be used for the treatment of patients with SARS-CoV-2 who have not yet developed cytokine storms. The changes in host factors regulated by the depletion of METTL3 and the specific mechanisms affecting viral replication need to be further studied.

Mohr et al. revealed that METTL3 activity contributes to the early steps of the SARS-CoV-2 replication cycle [108]. The inhibition of METTL3-mediated catalysis decreases sgRNA synthesis and viral N protein expression. The replication of SARS-CoV-2 can be inhibited by METTL3 depletion, treatment with a highly specific small-molecule inhibitor of METTL3, or YTHDF1/3 depletion. These results indicate that targeting m^6^A to limit SARS-CoV-2 replication may also be a therapeutic strategy.

### m**^6^**A in severe fever with thrombocytopenia syndrome virus

Severe fever with thrombocytopenia syndrome (SFTS) is an acute infectious disease caused by a new bunyavirus. Due to its high fatality rate and potential for a global pandemic, the World Health Organization declared SFTS as one of the ten top priority infectious diseases. At present, the main clinical treatment for SFTS is the broad-spectrum antiviral drug ribavirin. Zhou et al. performed m^6^A-seq of clinical residual blood samples from patients with SFTS and found that patients infected with severe fever with thrombocytopenia syndrome virus (SFTSV) exhibit significant changes in the abundance of m^6^A compared with healthy individuals [109]. The genes containing these differential m^6^A peaks are mainly enriched in platelet development, viral transcription, type I interferon signaling, the immune response, and other pathways. Interestingly, m^6^A peaks are also found on the viral genome, which indicates that m^6^A may also exist in SFTSV. RNA sequencing (RNA-seq) was used to analyze the differential gene expression between SFTSV-positive and SFTSV-negative patients, and the results revealed that the *FTO* expression level in positive samples is higher than that in negative samples. As the disease symptoms improves and the viral copy number decreases, the expression of *FTO* decreases slowly. These findings suggest that some immunomodulatory genes may be regulated by m^6^A after SFTSV infection. Future studies, such as experiments with cell models, are needed to explore the relationship between m^6^A and SFTSV.

## Perspectives

As one of the most common modifications of RNA, m^6^A participates in many important biological processes. Although m^6^A was discovered in a variety of viruses more than 40 years ago, the relationship between m^6^A and viruses was unclear. With the development of detection technology, the mechanism by which m^6^A-related proteins regulate viral replication and other processes has been gradually revealed in recent years. For example, host cells actively respond to viral infection by impairing the demethylation activity of ALKBH5 and thereby reprogramming the cellular metabolic state [110]. By recognizing m^6^A and binding to m^6^A-containing viral RNA, the reader protein YTHDF affects the processing of RNA and then regulates viral activity and infection [93]. METTL3 interacts with viral RNA-dependent RNA polymerase 3D to enhance the stability and transcription efficiency of 3D protein by increasing the ubiquitination modification level of 3D, which results in the promotion of viral replication [19].

m^6^A may exert completely opposite regulatory effects on different viruses. For example, m^6^A may positively regulate SV40 and HIV-1 and negatively regulate HBV, ZIKV, and HCV. The same virus, which may infect different host cell types, may also show opposite results depending on the cell type, *e.g.*, KSHV. In general, m^6^A is neither uniformly antiviral nor proviral but rather regulates viral production by affecting specific RNAs. The reasons for these differences remain unclear, and the current understanding of the regulatory mechanism of m^6^A in viral infections is extremely limited; this question thus needs to be explored in future studies. Methyltransferase inhibitors or demethylase inhibitors can regulate the m^6^A content and thus regulate the life cycle or infection efficiency of a virus, and these agents are expected to be effective antiviral drugs.

We expect that future studies provide a better understanding of the impact of m^6^A on life cycle processes such as viral replication and provide fundamental research support for the development of drugs to treat viral diseases. The mechanism by which m^6^A regulates the stability, shearing, and translation of RNA transcripts needs to be further studied. The development of technology for the detection of m^6^A with a low input is also urgently needed. Some existing viruses, such as SFTSV, cannot currently be treated with a specific drug or prevented by a vaccine, and modified nucleotides play an important role in mRNA vaccines. Whether m^6^A could play a role in vaccine development is also worth exploring.

## Competing interests

Both authors have declared no competing interests.

## CRediT authorship contribution statement

**Yafen Wang:** Conceptualization, Investigation, Writing – original draft, Visualization. **Xiang Zhou:** Conceptualization, Writing – review & editing. Both authors have read and approved the final manuscript.
